# Evaluation of sexual function and vaginal prolapse after radical cystectomy in women: a study to explore an under-evaluated problem

**DOI:** 10.1007/s00192-023-05611-4

**Published:** 2023-08-15

**Authors:** Maren Juliane Wenk, N. Westhoff, B. Liedl, M. S. Michel, B. Grüne, M. C. Kriegmair

**Affiliations:** 1 Department of Urology and Urological Surgery, University Medical Center Mannheim, University of Heidelberg, Theodor-Kutzer-Ufer 1–3, 68167 Mannheim, Germany; 2 Center of Reconstructive Urogenital Surgery, Urologische Klinik Planegg, Germeringer Str. 32, 82152 Planegg, Germany

**Keywords:** Female sexual function, Sexual activity, Quality of life, Vaginal prolapse, Radical cystectomy in women, Patient-reported outcome measures

## Abstract

**Introduction and hypothesis:**

The objective was to evaluate sexual function, vaginal prolapse, and quality of life (QoL) in women after radical cystectomy (RC) using validated questionnaires and pelvic organ prolapse quantification (POP-Q) measurement.

**Methods:**

Female bladder cancer patients who underwent RC at our tertiary care center were included (January 2008 to March 2022). Patients received three validated questionnaires (International Consultation on Incontinence Questionnaire Vaginal Symptoms [ICIQ-VS] Part A, Pelvic Organ Prolapse/Urinary Incontinence Sexual Questionnaire IUGA revised [PISQ], European Organization for Research and Treatment of Cancer Quality of Life Questionnaire [EORTC] C30/BLM30). Patients who consented were examined with vaginal POP-Q measurement.

**Results:**

Out of 322 patients, 193 were still alive, 54 patients were lost to follow-up, and 43 were excluded, resulting in 96 patients who received the questionnaire. Finally, 35 patients were included, of whom 17 patients consented to vaginal examination. Complaints due to vaginal symptoms were low (ICIQ-VS 6.17 + 5.37). Sexual activity was reported by 12 patients (34.3%); 23 patients (65.71%) were not sexually active. No apical prolapse was found in POP-Q measurement; 6 patients (35.3%) had anterior, and 14 patients (82.4%) posterior prolapse; the highest prolapse stage was 2. No significant differences were found regarding POP stages, sexual function, and QoL (all *p* > 0.05) when comparing continent and incontinent urinary diversions. Comparing the vaginal approach (no sparing vs sparing), significant differences were found in only two PISQ subscales (significantly higher scores after vagina sparing, *p* = 0.01 and *p* = 0.02).

**Conclusions:**

The type of urinary diversion, POP-Q stages, and tumor stages did not show significant differences regarding sexual function, QoL, and prolapse complaints in women after RC, whereas a vagina- sparing approach showed significant differences only in two subscales without clinical relevance.

**Supplementary information:**

The online version contains supplementary material available at 10.1007/s00192-023-05611-4

## Introduction

In Germany, approximately 18,270 people developed invasive bladder cancer in 2018, a quarter of whom were women (https://www.krebsdaten.de/Krebs/DE/Content/Krebsarten/Harnblasenkrebs/harnblasenkrebs_node.html). Although the ratio of male to female is 4:1, women tend to have more aggressive disease requiring radical management [1]. In nonmetastatic muscle-invasive bladder cancer, radical cystectomy (RC) with pelvic lymph node dissection is the gold standard therapy. In women, the proximity of the vagina and uterus to the bladder carries the risk of adjacent spread of cancer. Therefore, RC is often performed as part of an anterior exenteration with concurrent removal of the bladder, uterus, and anterior wall of the vagina [2]. Generally, the Fallopian tubes and ovaries are removed as well. The excision of the anterior vaginal wall may lead to vaginal tightness and dyspareunia postoperatively; the removal of the ovaries can cause hormonal changes and decreased vaginal lubrication [3]. Additionally, the disruption of the pelvic nerve plexus running along the lateral vagina, as well as the possible devascularization of the clitoris during urethrectomy may significantly change female sexual function after RC [4].

Nowadays, the improvement of cancer-related life expectancy allows an increased focus on quality of life (QoL) [5], but little attention has been directed toward female sexual function after RC [4]. Recent data suggest that preservation of the genital or pelvic organs in women yields better sexual outcomes than RC without compromising oncological outcomes in well-selected patients, but that none of these techniques can be recommended over the classical standard RC [6].

However, RC with a vaginal or genital organ-sparing approach may be associated with complications specific to the female population such as pelvic organ prolapse (POP), vaginal dehiscence or evisceration, and dyspareunia [7]. The problem of vaginal prolapse after RC has hardly been investigated to date. Although case series of these individual complications have been reported, the actual incidence remains unknown [7, 8]. Past studies indicated that vaginal prolapse after RC is probably an underestimated problem that can severely affect the QoL of women postoperatively [9]. As the focus has been on oncological cure and therapy, the diagnosis and treatment of vaginal prolapse may be delayed or even forgotten [10].

To our knowledge, patient-reported outcome measures (PROMS) after RC regarding vaginal prolapse, as well as structured vaginal examination, have not yet been systematically evaluated in a study before. Therefore, the aim of our study is to evaluate subjective outcomes such as sexual function, vaginal prolapse symptoms, and QoL in women at least 6 months after RC using validated questionnaires and the objective outcome of a vaginal prolapse measured by pelvic organ prolapse quantification (POP-Q) measurement. Hopefully, the study will provide more accurate information on the prevalence and prolapse-related limitations of female RC patients in daily life.

Therefore, the aim of our study is to evaluate the subjective outcomes such as sexual function, vaginal prolapse symptoms, and QoL in women at least 6 months after RC using validated questionnaires and the objective outcome of vaginal prolapse measured by the POP-Q.

## Patients and methods

### Study population and data collection

After Institutional Ethical Approval (No. 2021–596) was granted, all female patients undergoing RC for bladder cancer at our tertiary care hospital from 1 January 2008 to 30 March 2022, were identified using the Operation and Procedure Codes for RC. A cross-sectional explorative cohort study was performed. These patients were contacted if RC had been performed more than 6 months ago. In addition to the questionnaires, they were invited to undergo a voluntary vaginal examination with evaluation of POP-Q stages. Exclusion criteria were previous radiation of the pelvis, a previous gynecological tumor, and patients with malformations and non-oncological cystectomies.

### Questionnaires

All included patients completed three validated questionnaires assessing prolapse symptoms, QoL, and sexual symptoms: the German version of the International Consultation on Incontinence Questionnaire Vaginal Symptoms Part A (ICIQ-VS Part A) [11], the German version of the Pelvic Organ Prolapse/Urinary Incontinence Sexual Questionnaire (PISQ) International Urogynecological Association (IUGA) Revised (PISQ-IR) [12], and the German version of the European Organization for Research and Treatment of Cancer Quality Of Life Questionnaire Muscle-Invasive Bladder Cancer Module (EORTC QLQ C30 and BLM 30) [13]. Scoring, interpretation, and calculation in the case of missing values for all three questionnaires were performed as suggested by the authors.

The ICIQ-VS Part A evaluates a comprehensive range of vaginal symptoms and their impact on quality of life, particularly those of POP. It contains nine questions. The symptom score ranges from 0 to 53 with higher scores indicating a high level of symptomatology and therefore a high level of complaints.

The PISQ-IR evaluates sexual function for women with POP and urinary and/or fecal incontinence. It contains five questions in the case of sexual inactivity (NSA) and 14 questions in the case of sexual activity (SA). Symptom scores for NSA patients are assessed on four independent scales: partner-related (NSA-PR), condition-specific (NSA-CS), global quality (NSA-GQ), and condition impact (NSA-CI). Higher scores in symptom scales indicate a higher level of problems; scores range from 1.0 to 4.0 (PR, CS, CI) and from 1.0 to 4.5 (GQ) respectively. The results for SA patients are presented as a sum score (range 1.0–4.62) with higher scores indicating better sexual function.

The EORTC QLQ–C30 evaluates QoL in cancer patients; the BLM 30 module evaluates QoL specifically in bladder cancer patients. It originally contained 60 questions, but two questions were deleted as they are directed at men only. All scales and single-item measure scores range from 0 to 100. High scores in functional scales indicate a high (healthy) level of functioning. A high score in QoL implies a high QoL. High scores in symptom scales/items indicate a high level of symptomatology (problems).

### Parameters

The following parameters were retrospectively collected from the patients’ electronic medical records: Age, body mass index (BMI), parity, American Society of Anesthesiologists (ASA) score, time since RC, chronic obstructive pulmonary disease (COPD), smoking history, history of POP, treatment of POP before and after RC, and hysterectomy before RC. In addition, the following perioperative parameters were recorded: blood loss, operation time, vagina-sparing technique, length and width of the intraoperatively resected vaginal tissue as indicated in the pathology report, urinary diversion, histology, and complications until first discharge (Clavien–Dindo Classification and Comprehensive Complication Index [CCI]).

### Surgical technique

In the case of a continent urinary diversion, the remaining vagina was fixed to the round ligament of the uterus and the greater omentum was placed between the neobladder/pouch and the vagina. In the case of incontinent urinary diversion, no specific fixation was performed. All patients in the study underwent open radical cystectomy. The procedures were performed by five different surgeons.

### Examination

The diagnosis of POP can only be made after a vaginal examination. Vaginal POP-Q measurement was performed in all patients who were willing to undergo vaginal examination. In patients with urinary diversions other than a neobladder, the urethral meatus is resected during RC and therefore not available as a landmark for POP-Q measurement. In these patients, the distal-most tip of the scar was used as the meatus measurement point for genital hiatus (GH) measurement. In addition, patients were asked about vaginal dryness during the examination. In this case, a vaginal estrogen was prescribed.

### Statistical analysis

Descriptive statistics were carried out to present the baseline and perioperative characteristics. Quantitative data were expressed as mean and standard deviation (SD) or median and interquartile range (IQR), and categorical data were expressed as absolute and relative frequencies. All statistical analyses were performed using JMP v14 (SAS Institute, Cary, NC, USA). Group comparison was performed using Pearson’s Chi-squared test or Fisher’s exact test (if necessary) for categorial variables and the Wilcoxon Mann–Whitney test for ordinal scaled variables. The level of significance was set at *p* = 0.05.

## Results

### Patient enrolment

**Fig. 1:**
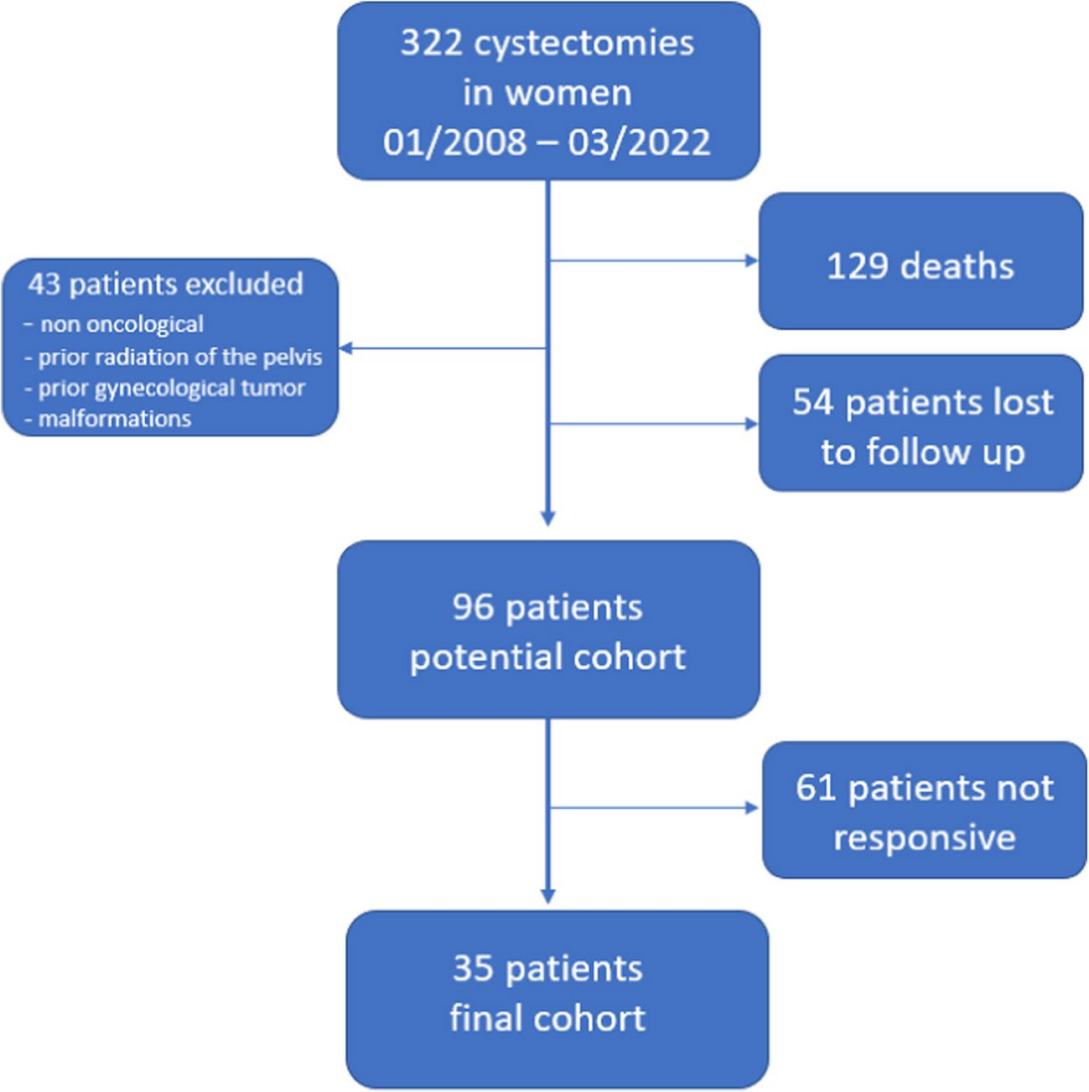
Patient enrolment flow diagram

From 01/2008 to 09/2022, a total of 322 RCs were performed in women for bladder cancer in our hospital. During follow-up, 129 patients died, 54 patients were lost to follow-up and 43 patients had to be excluded according to the exclusion criteria. Finally, 96 female patients received the questionnaire. Thirty-five patients responded, resulting in an overall response rate of 36.5%. Of those 35 patients, 17 agreed to vaginal examination and POP-Q measurement. Patient enrolment is shown in Fig. 1.

### Baseline characteristics

The mean age of the patients was 65.89 ± 9.05 years. 88.57% of the patients had given birth at least once, and an average of 2.1 + 0.93 children was reported, most of whom were delivered vaginally. Five patients (14.29%) reported POP treatment prior to RC, 10 patients (28.57%) had undergone hysterectomy prior to RC. None of the patients reported undergoing fixation surgery after cystectomy owing to prolapse. Baseline data are summarized in Table 1.

**Table 1:** Baseline demographics

Variable		Data
BMI (kg/m^2^), mean ± SD		26.52 ± 7.51
Age (years), mean ± SD		65.89 ± 9.19
Parity (*n*), mean ± SD		2.10 ± 0.94
Time since RC (years), mean ± SD		2.6 ± 3.85
Prior births, *n* (%)	Vaginal	26 (74.3)
	Caesarean section	1 (2.86)
	Both	4 (11.43)
	None	4 (11.43)
COPD, *n* (%)		3 (8.57)
Constipation, *n* (%)		13 (37.14)
Active smoking in the past 10 years, *n* (%)		12 (34.29)
Urinary incontinence prior to RC, *n* (%)	Yes	4 (11.43)
	I don’t remember	3 (8.57)
Hysterectomy prior to RC, *n* (%)		10 (28.57)
POP treatment prior to RC, *n* (%)	Yes	5 (14.29)
	Medication	2 (5.71)
	Surgery	1 (2.86)
	Physiotherapy	2 (5.71)
	I don’t remember	3 (8.57)

Demographics are expressed as mean and standard deviation, categorial variables as absolute and relative frequencies

*BMI* body mass index, *COPD* chronic obstructive pulmonary disease, *POP* pelvic organ prolapse, *RC* radical cystectomy

The mean operation time was 231.45 ± 90.96 min, and the average blood loss was 729.31 ± 385.80 ml. Twelve patients (34.29%) underwent early RC with a known tumor stage < T2. Six patients (17.14%) underwent neoadjuvant chemotherapy owing to advanced local growth and 5 patients (14.29%) received adjuvant treatment according to the recommendations of the bladder cancer guideline. Urethrectomy was performed in 23 patients (65.71%). A vagina-sparing approach was documented in 6 patients (17.14%) at the patient's request, and no organ sparing was performed. The mean length and width of the resected vaginal tissue were 4.75 ± 1.69 cm and 3.60 ± 1.33 cm respectively. Continent and incontinent urinary diversions were balanced (48.57% vs 51.43%). Regarding tumor stage, 54.29% had stage ≤ T1, whereas 45.71% had stage ≥ T2. Most patients had negative lymph nodes (N0 in 82.86%) and negative surgical margins (R0 in 97.14%). None of the patients had known distant metastases at the time of surgery. Complications of any type according to CDC occurred in 27 patients (77.14%), but only 4 patients (11.43%) had major complications ≥ grade III. The mean CCI was 19.77 ± 19.21. Cancer-specific characteristics are summarized in Table 2.

**Table 2:** Cancer-specific and operation-specific demographics of the study population

Variable	Data
ASA Score, median (IQR)	2.00 (2.00–2.00)
Charlson comorbidity index, mean ± SD	2.46 ± 1.36
Early cystectomy, *n* (%)	12 (34.29)
Operation time (min), mean ± SD	231.45 ± 90.96
Blood loss (ml), mean ± SD	729.31 ± 385.80
Neoadjuvant chemotherapy, *n* (%)	6 (17.14)
Adjuvant chemotherapy, *n* (%)	5 (14.29)
Vagina-sparing technique, *n* (%)	6 (17.14)
Resected vaginal tissue	*n* = 23
Length (cm), mean ± SD	4.75 ± 1.69
Width (cm), mean ± SD	3.60 ± 1.33
Urinary diversion	*n* = 35
Neobladder, *n* (%)	12 (34.29)
Pouch, *n* (%)	5 (14.29)
Ileum conduit, *n* (%)	17 (48.57)
Ureterostomy, *n* (%)	1 (2.86)
Histology (TNM), *n* (%)	*n* = 35
≤ T1	19 (54.29)
≥ T2	16 (45.71)
N0	29 (82.86)
N +	6 (17.14)
M0	35 (100.00)
R0	34 (97.14)
R1	1 (2.86)
Complications, *n* (%)	*n* = 35
Clavien–Dindo I	5 (14.29)
Clavien–Dindo II	18 (51.43)
Clavien–Dindo III a	2 (5.71)
Clavien–Dindo IV a	2 (5.71)
Comprehensive complication index, mean ± SD	19.77 ± 19.21

Data are expressed as mean and standard deviation or median and interquartile range, categorial variables as absolute and relative frequencies

*ASA* American Society of Anesthesiologists, n = number, *IQR* interquartile range, *SD* standard deviation*T* tumor stage, *N* lymph node involvement, *M* metastatic spread, *R* tumor margins

Four patients (11.43%) reported a feeling of bulging in the vagina after RC. The mean score of the ICIQ-VS Part A questionnaire was 6.17 ± 5.37. Therefore, overall complaints due to vaginal symptoms were low. The question with the most complaints in our cohort was 7a (“Do you feel that your vagina is too dry”).

In the PISQ-IR questionnaire, 12 patients (34.29%) reported to be SA, and 23 patients (65.71%) were NSA. The median sum score for SA patients was 3.79 (3.60–4.14). Partner-related reasons had the highest impact for why patients were not sexually active (2.50 [2.50–4.00]), whereas condition impact was rated the lowest (1.33 [1.00–2.17]). The results of all PISQ-IR scores can be seen in Table 3.

**Table 3:** Results of the questionnaires (German version of the International Consultation on Incontinence Questionnaire (*ICIQ*) Vaginal Symptoms (*VS*) Part A (*ICIQ-VS Part A*), German version of the Pelvic Organ Prolapse/Urinary Incontinence Sexual Questionnaire (*PISQ*) International Urogynecological Association (*IUGA*) Revised (*PISQ*), and the German version of the European Organization for Research and Treatment of Cancer (*EORTC*) Quality Of Life Questionnaire (*QLQ*) Muscle-Invasive Bladder Cancer Module (*EORTC-QLQ C30 and BLM 30*)

Questionnaire		Data
ICIQ–VS Part A, mean ± SD		
Score (*n* = 35)		6.17 ± 5.37
PISQ-IR, median (range)		
Sexually active (*n* = 12); sexually inactive (*n* = 23)	Sum score	3.79 (3.60–4.14)
	NSA-PR	2.50 (2.50–4.00)
	NSA-CS	2.00 (1.17–2.17)
	NSA-GQ	2.00 (1.33–2.75)
	NSA-CI	1.33 (1.00–2.17)
EORTC-QLQ C30, median (range)		
Global health QoL		66.67 (41.67–83.33)
Functional scales	Physical	73.33 (46.67–93.33)
	Role	66.67 (33.33–83.33)
	Emotional	58.33 (33.33–83.33)
	Cognitive	83.33 (66.67–100.00)
	Social	66.67 (50.00–100.00)
Symptom scales/items	Fatigue	44.44 (22.22–66.67)
	Nausea	0.00 (0.00–16.67)
	Pain	8.33 (0.00–37.5)
	Dyspnea	33.33 (0.00–66.67)
	Insomnia	66.67 (0–100.00)
	Appetite	0.00 (0.00–33.33)
	Constipation	0.00 (0.00–33.33)
	Diarrhea	0.00 (0.00–33.33)
	Finance	0.00 (0.00–33.33)
EORTC-QLQ BLM30, median (range)		
Multi-item scales	Urinary symptoms and problems	45.24 (30.95–55.95)
	Urostomy problems	27.78 (11.11–50.00)
	Future perspective	44.44 (22.22–77.78)
	Abdominal bloating and flatulence	33.33 (0.00–50.00)
	Body image	44.44 (22.22–66.67)
Single-item scales	Sexual functioning	0.00 (0.00–77.78)
	Catheter use problem	0.00 (0.00–33.33)

Results are expressed as mean and standard deviation or median and interquartile range

*SA* sexually active, *NSA* not sexually active, *NSA-PR* partner-related, *NSA-CS* condition specific, *NSA-GQ* global rating of sexual quality, *NSA-CI* condition impact, *QoL* quality of life

In addition, the results of the EORTC-QLQ C30 and BLM questionnaire are summarized in Table 3. In terms of global health QoL, patients scored a median of 66.67 (41.67–83.33). The “cognitive” scale was scored highest, with a median of 83.33 (66.67–100.00), followed by the scales “physical,” “social,” and “role.” Higher scores in symptom scales indicate a high level of problems; the scale with the highest scoring was “insomnia” with a median of 66.67 (0–100.00), followed by “fatigue,” “dyspnea,” and “pain.” Regarding bladder cancer-specific concerns, “urinary symptoms,” “future perspective,” and “body image” were rated highest (45.24, 44.44, and 44.44 respectively). “Sexual functioning” and “catheter use problems” appeared to be of low importance (both scored with a median of 0.00).

Seventeen patients consented to vaginal examination and POP-Q measurement. The results of POP-Q measurement are presented in Table 4. The total vaginal length (TVL) after RC was 7.41 ± 1.41 cm. None of the patients was diagnosed with apical prolapse. Anterior prolapse was found in 6 patients (35.29%), and the highest prolapse stage found was 2. One patient had postoperative narrowing of the vaginal introitus to 0.50 cm in width and therefore could not be vaginally examined (N.A.). Posterior prolapse was found in 14 patients (82.35%), the highest prolapse stage found was also 2. None of the patients expressed a desire for surgical prolapse correction owing to her complaints. One patient presented with a mass growing in the area of the former meatus urethrae, which was further investigated and histologically found to be a benign cyst.

**Table 4:** Pelvic organ prolapse (*POP*) stages

Variable	*N* = 17
Genital hiatus (cm), mean ± SD	2.56 ± 0.75
Perineal body (cm), mean ± SD	3.88 ± 0.86
Total vaginal length (cm), mean ± SD	7.41 ± 1.41
POP anterior, *n* (%)	
Stage 0	10 (58.82)
Stage 1	3 (17.65)
Stage 2	3 (17.65)
N.A.	1 (5.88)
POP posterior, *n* (%)	
Stage 0	2 (11.76)
Stage 1	7 (41.18)
Stage 2	7 (41.18)
N.A.	1 (5.88)

Data are expressed as median and interquartile range, categorial variables as absolute and relative frequencies

*IQR* interquartile range, *N.A.* not applicable, *SD* standard deviation

A subgroup analysis was performed to compare patients with incontinent (*n* = 18) and continent (*n* = 17) urinary diversions regarding cancer-specific demographic data, as well as questionnaire results and POP-Q measurements (Table 5). Interestingly, no significant differences were found for any of the variables (all *p* > 0.05).

**Table 5:** Pelvic organ prolapse (*POP*) stages, sexual function, and quality of life depending on urinary diversion

Variable	Incontinent	Continent	*p*
	*n* = 18	*n* = 17	
Age, median (range)	63.50 (58.25–74.25)	67.00 (59.50–72.50)	0.89
ASA Score, median (range)	2.00 (2.00–3.00)	2.00 (2.00–2.00)	0.16
Charlson Comorbidity Index, median (range)	3.00 (2.00–3.25)	2.00 (1.00–3.00)	0.19
Operation time (min), median (range)	255.50 (137.75–273.00)	216.00 (189.50–300.00)	0.43
Blood loss (ml), median (range)	650.00 (275.00–1075.00)	700.00 (500.00–1000.00)	0.71
Comprehensive Complication Index, median (range)	20.90 (8.70–30.58)	20.90 (0.00–20.90)	0.26
TVL (cm)	6.50 (5.13–8.75)	8.00 (7.00–9.00)	0.15
Length resected vaginal tissue (cm), median (range)	5.10 (4.00–6.10)	4.10 (3.13–5.50)	0.18
Vagina sparing, *n* (%)			1.00*
Yes	3 (16.70)	3 (17.60)	
No	15 (83.30)	14 (82.40)	
Tumour stage, *n* (%)			0.23**
≤ T1	8 (44.44)	11 (64.70)	
≥ T2	10 (55.56)	6 (35.30)	
ICIQ-VS Part A, median (range)			
	5 (0–8.5)	6 (3–9)	0.31
PISQ, *n* (%)			
Sexually active, median (range)	*n* = 9	*n* = 3	
	4.06 (3.76–4.63)	3.71 (3.15–3.86)	0.17
Sexually not active, median (range)	*n* = 20	*n* = 3	
NSA-PR, median (range)	2.75 (2.50–4.00)	2.50 (2.50–4.00)	0.86
NSA-CS, median (range)	2.00 (1.00–2.33)	2.00 (1.83–2.25)	0.50
NSA-GQ, median (range)	1.88 (1.31–2.56)	2.50 (1.50–3.13)	0.55
NSA-CI, median (range)	1.33 (1.00–2.00)	1.33 (1.00–2.33)	0.68
EORTC QLQ C30, median (range)	*n* = 18	*n* = 17	
Global health QOL	58.33 (16.67–79.17)	66.67 (50.00–83.30)	0.20
Physical	73.33 (41.67–80.0)	80.00 (63.33–93.33)	0.15
Role	66.67 (33.33–83.33)	66.67 (50.00–83.33)	0.43
Emotional	58.33 (16.67–75.00)	58.33 (37.50–87.50)	0.24
Cognitive	83.33 (58.33–100.00)	83.33 (66.67–100.00)	0.45
Social	66.67 (12.5–87.5)	66.67 (50.00–100.00)	0.67
Fatigue	44.44 (30.56–80.56)	33.33 (22.22–61.11)	0.33
Nausea	0.00 (0.00–58.33)	0.00 (0.00–0.00)	0.08
Pain	16.67 (0.00–50.00)	0.00 (0.00–33.33)	0.39
Dyspnea	33.33 (33.33–83.33)	33.33 (0.00–66.67)	0.13
Insomnia	66.67 (25.00–100.00)	33.33 (0.00–83.33)	0.52
Appetite loss	0.00 (0.00–41.67)	0.00 (0.00–33.33)	0.87
Constipation	0.00 (0.00–33.33)	0.00 (0.00–33.33)	0.74
Diarrhea	0.00 (0.00–41.67)	0.00 (0.00–33.33)	0.88
Finance	0.00 (0.00–66.67)	0.00 (0.00–33.33)	0.46
EORTC QLQ BLM 30, median (range)			
Urinary symptoms and problems	–	–	
Urostomy problems	27.78 (11.11–50.00)	27.78 (22.22–50.00)	0.76
Future perspective	61.11 (22.22–97.22)	33.33 (22.22–72.22)	0.66
Abdominal bloating and flatulence	16.67 (0.00–33.33)	50.00 (16.67–58.33)	0.18
Body image	55.56 (22.22–72.22)	33.33 (16.67–61.11)	0.44
Sexual functioning	0.00 (0.00–44.44)	33.33 (0.00–80.56)	0.31
Catheter use problem	–	–	
POP-Q, *n* (%)			
POP anterior	0.66		
Stage 0 anterior	4 (57.10)	6 (66.70)	
Stage 1 anterior	2 (28.60)	1 (11.10)	
Stage 2 anterior	1 (14.30)	2 (22.20)	
POP posterior, *n* (%)			0.55
Stage 0 posterior	1 (14.30)	1 (11.10)	
Stage 1 posterior	4 (57.10)	3 (33.30)	
Stage 2 posterior	2 (28.60)	5 (55.60)	

If not otherwise classified, all data are expressed as median and interquartile range

*ASA* American Society of Anesthesiologists, *RC* radical cystectomy, *SD* standard deviation, *TVL* total vaginal length, *ICIQ-VS Part A* International Consultation on Incontinence Questionnaire Vaginal Symptoms Part A, *PISQ* Pelvic Organ Prolapse/Urinary Incontinence Sexual Questionnaire, *SA* sexually active, *NSA* not sexually active, *NSA-PR* partner related, *NSA-CS* condition specific, *NSA-GQ* global rating of sexual quality, *NSA-CI* condition impact, *EORTC-QLQ C30/BLM* the European Organization for Research and Treatment of Cancer Quality Of Life Questionnaire/Muscle-Invasive Bladder Cancer Module, *QoL* quality of life *POP-Q* pelvic organ prolapse quantification

*Fisher’s exact test, **Pearson’s Chi-squared test

In another subgroup analysis tumor stages were compared (≤ T1, *n* = 19 vs ≥ T2, *n* = 16; Supplementary Table 1). The only variable that differed significantly between the two groups was the EORTC-QLQ C30 symptom “dyspnea,” which was rated significantly higher and thus worse in the ≥ T2 group (*n* = 0.002).

When comparing the groups regarding vagina sparing (no sparing *n* = 29 vs sparing *n* = 6), significant differences were found in the PISQ subscales NSA-GQ and NSA-CI (Supplementary Table 2). Patients who underwent a vagina-sparing approach reported significantly higher NSA-GQ and NSA-CI scores than patients who did not undergo vagina sparing (*p* = 0.01 and *p* = 0.02 respectively). In addition, significant differences were found regarding posterior prolapse in POP-Q measurement (*p* = 0.02).

## Discussion

In the present study, PROMS on sexual function, QoL, and vaginal prolapse after RC, as well as actual vaginal prolapse according to POP-Q measurement, were evaluated. Anterior and especially posterior vaginal prolapse were common, but prolapse stages were low, and symptoms not highly bothersome. The type of urinary diversion, the POP-Q stages, and the tumor stages did not show significant differences regarding sexual function, QoL, and prolapse complaints, whereas a vagina-sparing approach showed significant differences only in two subscales without clinical relevance.

### Sexual function and quality of life

A clear gender bias exists in the assessment of sexual outcomes for women undergoing urological surgery [4]. Women are known to have significantly worse sexual function scores after RC and female gender is an independent risk factor in a multivariate analysis [14], making it especially important that patients be appropriately educated. Although rates of sexual dysfunction > 65% have been reported across all cancer types [15], less than half of women undergoing RC receive preoperative counseling about the impact on sexual function [16–18]. Furthermore, only 17.3% of women undergoing RC actively asked their health care providers about postoperative sexual function [19], which underlines the importance of the surgeon specifically addressing the topic.

Westerman et al. reported that 53% of patients after RC (with a mean age of 68.5 years) had interest in sexual activity, and 40% endorsed sexual activity within the last 4 weeks—no gender differences were found [14]. This is consistent with our finding, as 34% of our study cohort reported being sexually active. Therefore, the surgeon should address the topic, assess sexual function in women systematically pre- and postoperatively, and female patients should be counseled accordingly.

Female sexual function after RC has been evaluated in a small number of studies to date, but only a small proportion used validated questionnaires (such as the Female Sexual Function Index [FSFI] questionnaire or the EORTC-QLQ BLM30) [4]. A study by Bhatt et al. found significantly better sexual function according to the FSFI in female patients after nerve-sparing RC, whereas no difference could be found for women who were SA and received vagina sparing (compared with no sparing) in our study cohort (*p* = 0.58) [20]. Interestingly, NSA women reported significant differences regarding the domains “global quality” and “condition impact” (*p* = 0.01 and *p* = 0.02 respectively) after vagina sparing. The cohort of vagina-sparing NSA patients was very small (*n* = 3), and 2 of these women were involuntarily NSA owing to progressive cancer and surgical complications leading to urinary incontinence, which explains the high GQ and CI scores. The results weigh more heavily because of the small number of patients. The fact that these women were involuntarily NSA is probably the reason for the worse scores compared with the "voluntarily" NSA patients in the nonsparing group.

All patients who received vagina sparing in our cohort had surgery no more than 2 years ago. This may indicate increasing awareness of female sexual function. In our cohort, there were no patients who received an organ-sparing procedure, which may be due to the high mean age of 65.89 years and the fact that almost 30% had undergone hysterectomy before RC. Although 54% of the patients were diagnosed with ≤ T1 stages in the cystectomy specimen, only 6 patients underwent surgery with a vagina-sparing approach. One must always keep in mind that the decision for vagina-sparing, or organ-sparing surgery is very individual. Many patients opt against improved function and for increased oncological safety. In addition, there is often uncertainty owing to imaging, as staging is often performed after transurethral resection and thus findings cannot be properly assessed (postoperative changes vs locally advanced tumor). Finally, 6 patients received neoadjuvant chemotherapy, which may have reduced tumor stage in the cystectomy specimen but was administered owing to advanced stage, so a vagina-sparing approach was probably not recommended for these patients.

Quality of life in women after RC is a topic of interest that has become even more important in the last decade. Studies mostly compare QoL regarding sex, urinary diversions, and different surgical approaches (especially nerve-sparing techniques). When comparing the results of patients with incontinent and continent urinary diversions in our study cohort, no significant differences were found in the EORTC questionnaire. This is in accordance with the findings of several authors in the past who found no significant diversion-related differences regarding QoL [21–23], whereas other studies have shown better results for either incontinent [24] or continent [25] diversions. Therefore, whether the type of urinary diversion really influences the rates of sexual function and QoL remains unclear [7, 21].

High BMI and COPD with chronic cough are known risk factors for vaginal prolapse. Even though the mean BMI in our study cohort was slightly overweight (26.52 kg/m^2^), the rates of bothersome prolapse complaints were low. Only very few patients reported having COPD (8.6%), which seems low given the fact that 35% had been actively smoking. Constipation was common, and patients who found it bothersome received laxative therapy after a personal consultation. Most patients who were examined for the study stated that they had never had a gynecological examination again after RC as all organs had been removed and further consultation was therefore considered unnecessary.

### Vaginal prolapse

Aside from oncological considerations, pre-existing POP in female RC patients should be fully assessed preoperatively and before counseling about urinary diversions and especially neobladder, which is hardly the case [8, 18]. The presence of POP contributes to hyper-continence and can lead to post-surgical pelvic floor disorders [26]. When evaluating vaginal prolapse after RC, mostly single-center case reports can be found, but only a few studies with mostly small collectives exist, all of which are retrospective. When comparing our results with those in the literature, it must be considered that there is great heterogeneity in terms of prolapse definitions and study designs and mostly, there are no clear definitions provided at all. A recent review found that symptoms of POP after RC were systematically assessed in 5 studies, but only 1 used a validated questionnaire and none of the studies included a validated objective measurement of POP [7]. Two studies did not report cases of postoperative prolapse [7], whereas 3 studies reported prolapse incidence ranging from 6 to 23% depending on the sample size and the time since RC [27–29]. It has to be mentioned that most studies were performed on women with a neobladder [7], whereas one study did not state the type of urinary diversion [29]. Another retrospective study reported that 17% of female RC patients were assessed for POP at any postoperative visit and found an incidence of 12% [30]. Vaginal prolapse was found in 35% (anterior) and 82% (posterior) of the patients who underwent POP-Q measurement in our study, which seems high at first consideration, but all patients had prolapse stages of ≤ 2 and none had the desire for surgical correction because of their prolapse symptoms. If anything, conservative therapies such as laxatives were sufficient for symptom relief. Interestingly, most case series report apical prolapse to be the predominant prolapse owing to disruption to the pelvic organ support structures, a finding that we could not confirm in our study as none of the patients was diagnosed with apical prolapse in POP-Q measurement.

The only study using a validated questionnaire to evaluate POP found that 22.9% of the patients reported the sensation of vaginal bulging [29], whereas this was reported by only 11.4% of our study cohort. These numbers are at odds with the fact that in a study by Swift, when asked, “Do you ever feel or see something bulging out of your vagina?,” 75% of patients with POP could be identified [31]. Thus, it must be said that vaginal sensation may be reduced after RC and therefore this question may not be reliable postoperatively. Even though they could not be included in the study, we know at least 3 female patients who had undergone RC and who presented to the gynecological center of our hospital with bothersome prolapse and underwent surgery. Unfortunately, they were not responsive to our questionnaire. Therefore, there may be a selection bias underestimating the real incidence of vaginal prolapse after RC, which highlights again the desperate need for prospective evaluation using standardized instruments and subjective outcome measures including vaginal examination. Our POP-Q measurement results cannot be compared with those of other female patients in the literature who had undergone RC, as this is the first study to perform structured and validated examination and measurement of vaginal prolapse in female patients who had undergone RC.

The following limitations of our study should be considered:

1. Our retrospective cohort size was small and the response rate not as high as expected. Therefore, no general conclusions can be drawn.
2. Not all questionnaires were validated specifically for RC patients.
3. There is a risk of selection bias by soliciting volunteers for the vaginal examination.

The strength of our study is that it is to our knowledge the first to use structured vaginal examination combined with validated questionnaires and PROMs after RC. Well-designed prospective studies with larger collectives are desperately needed to show the real incidence and degree of bother. Hopefully, this study will encourage further studies of female patients who have undergone RC to evaluate not only the surgical outcome but also the impact on women´s quality of life, the safety of vagina- and organ-sparing techniques, and potential associated complications in the future.

## Conclusion

This study is to our knowledge the first to use structured vaginal examination combined with validated questionnaires and PROMs to evaluate QoL, sexual function, and vaginal prolapse after RC. Even though anterior and especially posterior vaginal prolapse after RC were common, prolapse stages were low and symptoms not highly bothersome. The type of urinary diversion, the POP-Q stages, and the tumor stages did not show significant differences regarding sexual function, QoL, and prolapse complaints in validated questionnaires, whereas a vagina-sparing approach showed significant differences only in two subscales without clinical relevance. Participation was lower than expected and as vaginal examination was voluntary, results may underestimate the real prevalence and bother due to selection bias. This highlights the need for prospective studies, and especially the use of standardized instruments to evaluate the real incidence of vaginal prolapse and prolapse complaints after RC for bladder cancer in women. In addition, QoL, sexual function, and vaginal prolapse must be systematically assessed pre- and postoperatively, female patients should be counseled accordingly, and these specific issues should be addressed regularly during follow-up visits.

### Supplementary information

Below is the link to the electronic supplementary material.

Supplementary file1 (DOCX 27 KB)

## Data Availability

Original data of this study is available in case of justified request.

## Abbreviations

ASA: American Society of Anesthesiologists
BLM: Bladder cancer module
BMI: Body mass index
CCI: Comprehensive complication index
COPD: Chronic obstructive pulmonary disease
EORTC: European Organization for Research and Treatment of Cancer
GH: Genital hiatus
ICIQ-VS: International Consultation on Incontinence Questionnaire Vaginal Symptoms
IQR: Interquartile range
IUGA: International Urogynecological Association
N.A.: Not applicable
NSA-PR: Nonsexually active partner related
NSA-CS: Nonsexually active condition specific
NSA-GQ: Nonsexually active global quality
NSA-CI: Nonsexually active condition impact
PB: Perineal body
PISQ-IR: Pelvic Organ Prolapse/Urinary Incontinence Sexual Questionnaire IUGA Revised
POP: Pelvic organ prolapse
POP-Q: Pelvic organ prolapse quantification
PROMS: Patient-reported outcome measures
QLQ: Quality-of-life questionnaire
QoL: Quality of life
RC: Radical cystectomy
SA: Sexually active
SD: Standard deviation
TVL: Total vaginal length
