# Supplementary material for: Evaluation of sexual function and vaginal prolapse after radical cystectomy in women: a study to explore an under-evaluated problem
Source: Int Urogynecol J. 2023 Aug 15;34(12):2933–43. doi: 10.1007/s00192-023-05611-4 (PMC10756865; doi:10.1007/s00192-023-05611-4)
Supplement: Supplementary file 1 — Supplementary file1 (DOCX 27 KB) [file 192_2023_5611_MOESM1_ESM.docx]

**Supplementary material**

**Supplementary Table 1 Pelvic organ prolapse (POP) stages, sexual function, and quality of life depending on tumor stage**

| **Variable** | **< T1 (n = 19)** | **> T2 (n = 16)** | **p** |
| --- | --- | --- | --- |
| **Age** | 67.0 (59.00 – 74.00) | 63.50 (56.75 – 72.75) | 0.42 |
| **ASA-Score** | 2.00 (2.00 – 2.00) | 2.00 (2.00 – 3.00) | 0.60 |
| **Charlson Comorbidity Index** | 3.00 (2.00 – 3.00) | 2.00 (1.00 – 3.00) | 0.21 |
| **Operation time [min]** | 204 (149.00 – 275.00) | 263 (192.00 – 305.25) | 0.24 |
| **Blood loss [ml]** | 700.00 (500.00 – 1000.00) | 700.00 (350.00 – 1000.00) | 0.96 |
| **Comprehensive Complication Index** | 20.90 (0.00 – 27.05) | 20.90 (8.70 – 20.90) | 0.93 |
| **TVL [cm]** | 7.50 (5.25 – 8.50) | 7.50 (7.00 – 9.00) | 0.46 |
| **Length resected vaginal tissue [cm]** | 4.30 (3.00 – 6.00) | 4.75 (3.93 – 5.58) | 0.71 |
| **Urinary diversion** | | | 0.23^ꝉ^ |
| Continent (n, %) | 11 (57.90) | 6 (37.50) |  |
| Incontinent (n, %) | 8 (42.10) | 10 (62.50) |  |
| **Vaginal sparing** | | | 0.67^×^ |
| Yes (n, %) | 4 (21.00) | 2 (12.50) |  |
| No (n, %) | 15 (79.00) | 14 (87.50) |  |
| **ICIQ-VS Part A** | | | |
|  | 5 (3 – 8) | 5.5 (0.5 – 9.5) | 0.62 |
| **PISQ** | | | |
| Sexually active (SA) | 3.69 (3.15 – 3.86) | 4.08 (3.78 – 4.63) | 0.11 |
| Sexually not active (NSA) | | | |
| NSA-PR | 2.50 (2.50 – 4.00) | 3.00 (2.50 – 4.00) | 0.31 |
| NSA-CS | 2.00 (1.17 – 2.17) | 2.00 (1.25 – 2.75) | 0.65 |
| NSA-GQ | 1.75 (1.25 – 3.50) | 2.13 (1.75 – 2.69) | 0.64 |
| NSA-CI | 1.50 (1.00 – 2.42) | 1.33 (1.00 – 2.00) | 0.56 |
| **EORTC QLQ C30** | | | |
| Global health QOL | 66.67 (50.00 – 83.33) | 50 (41.67 – 83.33) | 0.49 |
| Physical | 73.33 (60.00 – 93.33) | 73.33 (46.67 – 85.00) | 0.55 |
| Role | 66.67 (50.00 – 83.33) | 66.67 (33.33 – 83.33) | 0.69 |
| Emotional | 58.33 (33.33 – 83.33) | 50.00 (27.08 – 66.67) | 0.21 |
| Cognitive | 83.33 (66.67 – 100.00) | 83.33 (41.67 – 95.83) | 0.16 |
| Social | 66.67 (50.00 – 100.00) | 66.67 (41.67 – 83.33) | 0.75 |
| Fatigue | 33.33 (22.22 – 77.78) | 52.78 (25.00 – 66.67) | 0.31 |
| Nausea | 0.00 (0 – 16.67) | 0.00 (0.00 – 58.33) | 0.47 |
| Pain | 0.00 (0.00 – 33.33) | 16.67 (0.00 – 50.00) | 0.78 |
| Dyspnoe | 0.00 (0.00 – 33.33) | 66.67 (33.33 – 100.00) | **0.002** |
| Insomnia | 33.33 (33.33 – 66.67) | 66.67 (0.00 – 100.00) | 0.50 |
| Appetite loss | 0.00 (0.00 – 66.67) | 0.00 (0.00 – 25.00) | 0.43 |
| Constipation | 0.00 (0.00 – 33.33) | 16.67 (0.00 – 33.33) | 0.55 |
| Diarrhoea | 0.00 (0.00 – 33.33) | 0.00 (0.00 – 33.33) | 0.63 |
| Finance | 0.00 (0.00 – 66.67) | 0.00 (0.00 – 33.33) | 0.32 |
| **EORTC QLQ BLM30** | | | |
| Urinary symptoms and problems | 38.10 (26.19 – 57.14) | 47.62 (42.86 – 52.38) | 0.71 |
| Urostomy problems | 22.22 (11.11 – 30.56) | 38.89 (22.22 – 50.00) | 0.15 |
| Future perspective | 33.33 (22.22 – 66.67) | 55.56 (22.22 -100.00) | 0.45 |
| Abdominal bloating and flatulence | 33.33 (0.00 – 50.00) | 25.00 (12.5 – 54.17) | 0.70 |
| Body Image | 44.44 (22.22 – 66.67) | 33.33 (11.11 – 66.67) | 0.74 |
| Sexual functioning | 0.00 (0.00 – 77.78) | 0.00 (0.00 – 62.50) | 0.61 |
| Catheter use problem | 0.00 (0.00 - 58.33) | 0.00 (0.00 – 33.33) | 0.80 |
| **POP-Q** | | | |
| **POP anterior** | | | 0.72^×^ |
| Stage 0 anterior (n, %) | 5 (62.50) | 5 (62.50) |  |
| Stage 1 anterior (n, %) | 1 (12.50) | 2 (25.00) |  |
| Stage 2 anterior (n, %) | 2 (25.00) | 1 (12.50) |  |
| **POP posterior** | | | 0.87^×^ |
| Stage 0 posterior (n, %) | 1 (12.50) | 1 (12.50) |  |
| Stage 1 posterior (n, %) | 4 (50.00) | 3 (37.50) |  |
| Stage 2 posterior (n, %) | 3 (37.50) | 4 (50.00) |  |

**Supplementary Table 1 POP-stages, sexual function, and quality of life depending on tumor stage**

Supplemetary Table 1 summarizes the demographics and the results of the questionnaires comparing tumor stages < T1 and > T2 . If not otherwise classified, all data are expressed as median and interquartile range.

**p-values**: **×** p-value for Fisher’s exact test, **ꝉ** p-value for Pearson’s Chi square test

**Abbreviations**: ASA = American Society of Anaesthesiologists, n = number, RC = radical cytectomy, SD = standard deviation, cm = centimeter, ml = mililiter, TVL = total vaginal length, ICIQ - VS Part A = International Consultation on Incontinence Questionnaire Vaginal Symptoms Part A, PISQ = Pelvic Organ Prolapse / Urinary Incontinence Sexual Questionnaire, NSA-PR = partner related, NSA-CS = condition specific, NSA-GQ = global rating of sexual quality, NSA-CI = condition impact, EORTC QLQ C30 / BLM = the European Organization for Research and Treatment of CancerQuality Of Life Questionnaire / Muscle-Invasive Bladder Cancer Module, Qol = quality of life, POP = pelvic organ prolapse, POP-Q = pelvic organ prolapse quanitification measurement

**Supplementary Table 2 Pelvic organ prolapse (POP) stages, sexual function, and quality of life depending on vaginal sparing**

| **Variable** | **No sparing (n = 29)** | **Sparing (n = 6)** | **p** |
| --- | --- | --- | --- |
| **Age** | 69.00 (59.50 – 74.00) | 60.00 (58.30 – 68.00) | 0.38 |
| **ASA-Score** | 2.00 (2.00 – 2.00) | 2.00 (1.75 – 3.00) | 0.85 |
| **Charlson Comorbidity Index** | 2.00 (1.50 – 3.00) | 3.00 (1.75 – 4.75) | 0.24 |
| **Operation time [min]** | 208.00 (149.00 – 275.00) | 265.50 (213.00 – 285.00) | 0.22 |
| **Blood loss [ml]** | 700.00 (450.00 – 1000.00) | 800.00 (500.00 – 1062.50) | 0.50 |
| **Comprehensive Complication Index** | 200.90 (8.70 – 27.90) | 4.35 (0.00 – 40.68) | 0.20 |
| **TVL [cm]** | 7.00 (5.75 – 9.00) | 8.00 (7.25 – 8.75) | 0.39 |
| **Length resected vaginal tissue [cm]** | 4.75 (3.80 – 6.03) | 3.90 (3.25 – 5.75) | 0.43 |
| **Urinary diversion** | | | 1.00^×^ |
| Continent (n, %) | 14 (48.30) | 3 (50.00) |  |
| Incontinent (n, %) | 15 (51.70) | 3 (50.00) |  |
| **Tumour stage** | | | 0.67^×^ |
| < T1 | 15 (51.72) | 4 (66.70) |  |
| ≥ T2 | 14 (48.28) | 2 (33.30) |  |
| **ICIQ-VS Part A** | | | |
|  | 5 (2 – 8) | 6 (4.5 – 10) | 0.43 |
| **PISQ** | | | |
| Sexually active (SA) | 3.76 (3.30 – 4.30) | 3.86 (3.76 – 3.86) | 0.58 |
| Sexually not active (NSA) | | | |
| NSA-PR | 3.00 (2.50 – 4.00) | 2.5 (1.0 – 2.5) | 0.08 |
| NSA-CS | 2.00 (1.00 – 2.25) | 2.0 (2.0 – 2.33) | 0.36 |
| NSA-GQ | 1.75 (1.27 – 2.50) | 4.0 (2.75 – 4.25) | **0.01** |
| NSA-CI | 1.17 ( 1.00 – 2.00) | 4.0 (1.67 – 4.0) | **0.02** |
| **EORTC QLQ C30** | | | |
| Global health QOL | 66.67 (43.75 – 83.33) | 62.50 (16.67 – 68.75) | 0.36 |
| Physical | 73.33 (46.67 – 86.67) | 86.67 (65.00 – 95.00) | 0.16 |
| Role | 66.67 (33.33 – 83.33) | 66.67 (25.00 – 100.00) | 0.75 |
| Emotional | 58.33 (33.33 – 79.17) | 29.17 (0.00 – 87.50) | 0.26 |
| Cognitive | 83.33 (66.67 – 100.00) | 83.33 (79.17 – 100.00) | 0.52 |
| Social | 66.67 (5.00 – 91.67) | 66.67 (0.00 – 100.00) | 0.79 |
| Fatigue | 44.44 (27.78 – 72.22) | 27.78 (0.00 – 69.44) | 0.21 |
| Nausea | 0.00 (0.00 – 25.00) | 0.00 (0.00 – 37.50) | 0.92 |
| Pain | 16.67 (0.00 – 33.33) | 0.00 (0.00 – 50.00) | 0.59 |
| Dyspnoe | 33.33 (0 – 66.67) | 16.67 (0.00 – 41.67) | 0.20 |
| Insomnia | 33.33 (0.00 – 83.33) | 83.33 (25.00 – 100.00) | 0.29 |
| Appetite loss | 0.00 (0.00 – 33.33) | 0.00 (0.00 – 66.67) | 0.83 |
| Constipation | 0.00 (0.00 – 33.33) | 0.00 (0.00 – 50.00) | 0.73 |
| Diarrhoea | 0.00 (0.00 – 33.33) | 0.00 (0.00 – 25.00) | 0.30 |
| Finance | 0.00 (0.00 – 33.33) | 0.00 (0.00 – 16.67) | 0.27 |
| **EORTC QLQ BLM30** | | | |
| Urinary symptoms and problems | 47.62 (33.33 – 54.76) | 38.10 (23.81 – 66.67) | 0.85 |
| Urostomy problems | 27.78 (11.11 – 45.83) | 61.11 (5.56 – 73.33) | 0.46 |
| Future perspective | 44.44 (22.22 – 77.78) | 50.00 (22.22 – 91.67) | 0.78 |
| Abdominal bloating and flatulence | 33.33 (0.00 – 50.00) | 3.33 (12.50 – 70.83) | 0.54 |
| Body Image | 38.89 (13.89 – 66.67) | 72.22 (30.56 – 100.00) | 0.07 |
| Sexual functioning | 0.00 (0.00 – 66.11) | 28.89 (0.00 – 86.11) | 0.50 |
| Catheter use problem | - | - |  |
| **POP-Q** | | | |
| **POP anterior** | | | 0.54 |
| Stage 0 anterior | 7 (58.30) | 3 (75.00) |  |
| Stage 1 anterior | 2 (16.70) | 1 (25.00) |  |
| Stage 2 anterior | 3 (25.00) | 0 (0.00) |  |
| **POP posterior** | | | **0.02** |
| Stage 0 posterior | 0 (0.00) | 2 (50.00) |  |
| Stage 1 posterior | 5 (41.70) | 2 (50.00) |  |
| Stage 2 posterior | 7 (58.30) | 0 (0.00) |  |

**Supplementary Table 2 POP-stages, sexual function, and quality of life depending on vaginal sparing**

Supplemetary Table 2 summarizes the demographics and the results of the questionnaires comparing vaginal sparing and no sparing technique . If not otherwise classified, all data are expressed as median and interquartile range.

**p-values**: **×** p-value for Fisher’s exact test, **ꝉ** p-value for Pearson’s Chi square test

**Abbreviations**: ASA = American Society of Anaesthesiologists, n = number, RC = radical cytectomy, SD = standard deviation, cm = centimeter, ml = mililiter, TVL = total vaginal length, ICIQ - VS Part A = International Consultation on Incontinence Questionnaire Vaginal Symptoms Part A, PISQ = Pelvic Organ Prolapse / Urinary Incontinence Sexual Questionnaire, NSA-PR = partner related, NSA-CS = condition specific, NSA-GQ = global rating of sexual quality, NSA-CI = condition impact, EORTC QLQ C30 / BLM = the European Organization for Research and Treatment of CancerQuality Of Life Questionnaire / Muscle-Invasive Bladder Cancer Module, Qol = quality of life, POP = pelvic organ prolapse, POP-Q = pelvic organ prolapse quanitification measurement
